# Natural and Synthetic Modulators of the TRPM7 Channel

**DOI:** 10.3390/cells3041089

**Published:** 2014-11-27

**Authors:** Vladimir Chubanov, Sebastian Schäfer, Silvia Ferioli, Thomas Gudermann

**Affiliations:** Walther Straub Institute of Pharmacology and Toxicology, University of Munich, Goethestrasse 33, 80336 Munich, Germany; E-Mails: sebastian.schaefer@daad-alumni.de (S.S.); silvia.ferioli@lrz.uni-muenchen.de (S.F.); thomas.gudermann@lrz.uni-muenchen.de (T.G.)

**Keywords:** TRPM7, TRPM6, TRP channel, α-kinase, magnesium, calcium

## Abstract

Transient receptor potential cation channel subfamily M member 7 (TRPM7) is a bi-functional protein comprising a TRP ion channel segment linked to an α-type protein kinase domain. Genetic inactivation of TRPM7 revealed its central role in magnesium metabolism, cell motility, proliferation and differentiation. TRPM7 is associated with anoxic neuronal death, cardiac fibrosis and tumor progression highlighting TRPM7 as a new drug target. Recently, several laboratories have independently identified pharmacological compounds inhibiting or activating the TRPM7 channel. The recently found TRPM7 modulators were used as new experimental tools to unravel cellular functions of the TRPM7 channel. Here, we provide a concise overview of this emerging field.

## 1. Functional Roles of TRPM7

**Figure 1:**
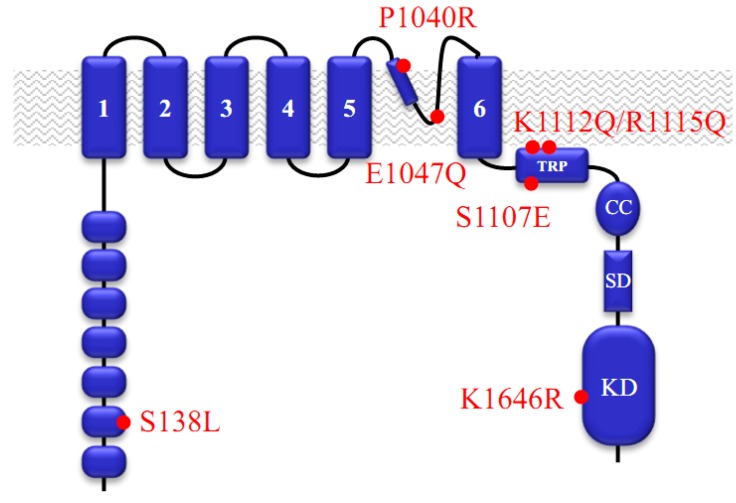
Domain topology of the murine kinase-coupled channel Transient receptor potential cation channel subfamily M member 7 (TRPM7).

TRPM7 is a plasma membrane protein that contains a transmembrane ion channel segment linked to a cytosolic α-type serine/threonine protein kinase domain as illustrated in Figure 1 [1,2,3,4,5]. It is commonly accepted that the overall architecture of the pore-forming segment of TRPM7 channels is analogous to that of tetrameric potassium channels. The channel domain of TRPM7 comprises six transmembrane helixes (Figure 1). A stretch of amino acids between 5th and 6th helices contains a predicted pore helix followed by a predicted pore loop (Figure 1). Like in potassium channels, it is assumed that the pore loops of four channel subunits contribute to a common ion selectivity filter. Among all ion channels, only TRPM7 and its homologous protein TRPM6 are known as channels covalently fused to kinase domains [1,6,7,8,9,10,11]. TRPM7 is a ubiquitously expressed protein and endogenous TRPM7 currents were detected in all cells investigated so far [12,13,14,15]. 

The plasma membrane channel segment of TRPM7 comprises six transmembrane helices (***1–6***). A short stretch between the 5 and 6 helices contains a predicted pore forming loop and pore helix [16]. A large cytosolic N-terminus of TRPM7 contains a set of domains that are highly conserved among the TRPM gene family and resemble ankyrin repeats as revealed by 3D modeling [11]. A C-terminus of TRPM7 contains a highly conserved transient receptor potential (***TRP***) domain, a coiled-coil (***CC***) domain, a kinase substrate domain (***SD***) and a kinase domain (***KD***). Red dots indicate the location of point mutations that were highly instrumental in probing of TRPM7 functions. Mutations **S138L** and **P1040R** correspond to TRPM6 missense mutations causing an inherited disorder in humans known as hypomagnesemia and secondary hypocalcemia (HSH) [9,17]. S138L disrupts assembly of TRPM7 channel complexes [9], whereas P1040R results in a dominant-negative channel subunit [17]. E1047 is a negatively charged residue located in a ‘selectivity’ filter of the TRPM7 channel pore and **E1047Q** mutation results in an active channel permeable to monovalent cations and impermeable to divalent ions like Ca^2+^ and Mg^2+^ [16,18] recapitulating the characteristic feature of the taste-signaling TRPM5 channel [19,20]. The **S1107E** mutation produces a constitutively active channel insensitive to intracellular Mg^2+^ and PIP_2_ [21]. Positively charged residues K1112 and R1115 were suggested to be required for PIP_2_ dependent gating of TRPM7 and, consequently, the **K1112Q/R1115Q** double mutation ablates TRPM7 currents [22]. K1646 is a highly conserved residue located in the catalytic site of the kinase domain [4] and the **K1646R** mutation is sufficient to block the kinase function of TRPM7 (‘kinase-dead’ mutation) [23,24].

Genetic ablation of TRPM7 in cultured cells revealed that TRPM7 regulates cellular Mg^2+^ levels [9,24,25,26], cell motility [27,28,29,30,31,32,33,34], proliferation/cell survival [1,24,26,35], differentiation [36,37], mechanosensitivity [28,38,39] and exocytosis [40]. Furthermore, it was suggested that TRPM7 plays a role in anoxic neuronal death [41], hypertension [42], neurodegenerative disorders [43,44], atrial fibrillation, cardiac fibrosis [45] and tumor growth/progression [46,47,48,49,50,51,52,53]. Genetic association studies in humans revealed that TRPM7 may be implicated in myocardial repolarization [54]. Experiments with *Trpm7* gene deficient mice and zebrafish and genetic association studies in humans showed that TRPM7 is required for early embryonic development [25,55,56,57], thymopoiesis [55], morphogenesis of the kidney [57], cardiac rhythmicity [58], cardiac repolarization [59] and systemic Mg^2+^ homeostasis [25] - though the latter finding remains controversial [55].

Our mechanistic understanding of the functional interplay between TRPM7 kinase and channel moieties is still in its infancy. In vitro, TRPM7 kinase is able to phosphorylate serine/threonine residues of annexin A1 [60], myosin II isoforms [61], eEF2-k [62] and PLCγ2 [63]. Furthermore, multiple residues located in a ‘substrate’ segment of TRPM7 are potential autophosphorylation targets of the kinase domain [64,65]. Recently, it was shown that the TRPM7 kinase domain can be cleaved by caspases during Fas-receptor stimulation in immune cells [66]. The truncated channel exhibited substantially higher activity and potentiated Fas-receptor signaling [66]. In another study, the cleaved TRPM7 kinase domain was found in multiple tissues and cell lines. The mechanism of TRPM7 cleavage was not established. Interestingly, the portion of TRPM7 containing the channel domain is eliminated, whereas the released kinase domain is able to translocate into the cell nucleus and phosphorylates histones to modulate the chromatin covalent modification landscape [67]. However, the physiological relevance of these findings remains to be elucidated. Along these lines, Kaitsuka *et al.* [23] have recently shown that mice carrying a point mutation in the catalytic site of the TRPM7 kinase domain (‘kinase-dead’ knock-in mutation, Figure 1) display an unaltered lifespan as well as normal Ca^2+^ and Mg^2+^ serum levels and do not develop obvious pathophysiologic phenotypes. 

The channel segment of TRPM7 forms a constitutively active ion channel that is highly selective for divalent cations such as Zn^2+^, Ca^2+^ and Mg^2+^ [1,2,67,68]. It has been hypothesized that influx of all these cations is relevant for the physiological role of TRPM7 [1,2,68]. Mutagenesis of the pore-forming sequence of TRPM7 allowed for the identification of specific residues that contribute to the ‘selectivity filter’ of the channel pore (Figure 1) [16,18]. In contrast, molecular mechanisms underlying TRPM7 channel gating are still a matter of debate. The prevailing models are mainly resting upon two findings. First, perfusion of cells with an Mg^2+^ free internal solution induces TRPM7 currents implying that intracellular Mg^2+^ (either free Mg^2+^ or Mg^2+^-ATP) may be a physiological negative regulator of the channel [1,69,70]. Experiments with the ‘kinase-dead’ knock-in mutation (Figure 1) or a channel variant lacking the whole kinase domain led to the concept that the kinase domain modifies the sensitivity of the TRPM7 channel to Mg^2+^ and Mg^2+^-ATP [24,69]. However, Hofmann *et al.* have shown recently that the TRP domain plays a key role in Mg^2+^ dependent gating of TRPM7 since a point mutation of a conserved serine residue in the TRP domain (Figure 1) is sufficient to create a constitutively active TRPM7 channel insensitive to intracellular Mg^2+^ [21].

The second model is predicated on the observation that the TRPM7 channel is tightly regulated by the plasma membrane phospholipid phosphatidylinositol 4,5-bisphosphate (PIP_2_) [71]. Consequently, stimulation of phospholipase C (PLC)-coupled G protein-coupled receptors (GPCRs) causes depletion of membrane PIP_2_ and, subsequently, inactivation of TRPM7 currents even in the absence of Mg^2+^ [71]. Kozak *et al.* [72] hypothesized that internal Mg^2+^ interacts directly with negatively charged PIP_2_ to interfere with the gating process of TRPM7. Recently, Xie *et al.* [22] reported that neutralization of basic residues in the TRP domain (Figure 1) leads to non-functional or dysfunctional TRPM7 with dampened regulation by PIP_2_ suggesting that the TRP domain may interact with PIP_2_.

## 2. Pharmacological Compounds Inhibiting the TRPM7 Channel

**Figure 2:**
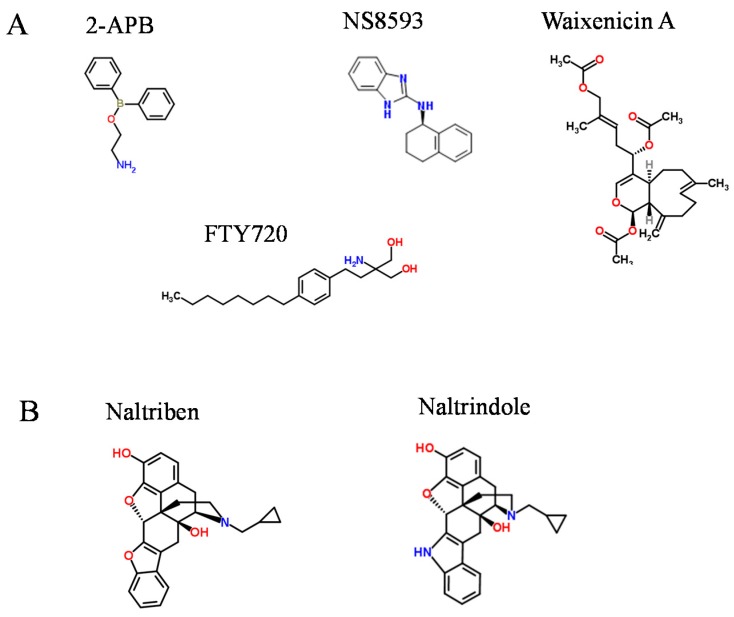
Chemical structures of modulators of the TRPM7 channel. (**A**) A subset of broadly used inhibitors of the TRPM7 channel; (**B**) A newly identified activator of the TRPM7 channel, naltriben, and the related inactive compound naltrindole.

Because of the pivotal role of the TRPM7 channel in physiology and pathophysiology, there is a pressing need to identify pharmacological compounds allowing to acutely probe TRPM7 channel *versus* kinase activity. Efforts of several laboratories resulted in the independent identification of an array of small organic compounds behaving as blockers of the TRPM7 channel as summarized in Table 1 and Figure 2a.

**Table 1:** Organic compounds inhibiting TRPM7 channel.

Compound	IC_50_ (μM) *	Description of the block	Reference
2-APB	174	Reversible	[73,74]
Spermine	2.3 ^†^	Reversible, voltage dependent	[75]
SKF-96365	n.d.	Tested only at 20 μM	[75]
Nafamostat	617	Reversible, voltage dependent	[76]
Carvacrol	306	Reversible	[77]
NDGA	n.d.	Tested only at 10 and 20 μM	[78]
AA861	n.d.	Tested only at 10 and 40 μM	[78]
MK886	n.d.	Tested only at 10 μM	[78]
Waixenicin A	7.0	Irreversible, [Mg^2+^]_i_ dependent	[79]
NS8593	1.6	Reversible, [Mg^2+^]_i_ dependent	[80]
Quinine	n.d	Reversible, tested only at 30 μM	[80]
CyPPA	n.d	Tested only at 30 μM	[80]
Dequalinium	n.d	Tested only at 30 μM	[80]
SKA31	n.d	Tested only at 30 μM	[80]
UCL 1684	n.d	Tested only at 30 μM	[80]
Sphingosine	0.6	Reversible	[81]
FTY720	0.7	Reversible	[81]

* IC_50_ values were shown for recombinant TRPM7 currents measured in the absence of internal Mg^2+^. **^†^** The dose-dependent effect of spermine was evaluated on endogenous TRPM7 currents in divalent-free external solution. n.d. - not determined.

The list of TRPM7 inhibitors comprises a group of non-specific channel blockers such as spermine, SKF-96365 and 2-aminoethyl diphenylborinate (2-APB), natural metabolites including waixenicin A, quinine and sphingosine and an array of drug-like synthetic compounds (Table 1). 2-APB (Figure 2a) reversibly blocked the endogenous TRPM7 channel in Jurkat T cells [73]. The inhibitory effect of 2-APB was characterized further with recombinant TRPM7 protein [74]. Extracellular spermine blocked endogenous TRPM7 currents in rat basophilic leukemia (RBL) cells with an IC_50_ value of 2.3 µM, and 20 µM SKF-96365 was sufficient for complete inactivation of TRPM7 in RBL cells [75]. It has been proposed that 2-APB does not act on TRPM7 directly, but rather inhibits the channel by means of intracellular acidification [82]. The broad spectrum serine protease inhibitor and anticoagulant nafamostat mesylate inhibited the TRPM7 channel with an IC_50_ of 617 µM [76]. Carvacrol [77] and several 5-lipoxygenase inhibitors (NDGA, AA861 and MK886) blocked TRPM7 currents in the high µM range [78].

Several small conductance Ca^2+^-activated K^+^ channel inhibitors such as the antimalarial plant alkaloid quinine, CyPPA, dequalinium, NS8593, SKA31 and UCL1684 also act as potent blockers of TRPM7 currents [80]. The most potent compound NS8593 (Figure 2a) inhibited the TRPM7 channel in an Mg^2+^-dependent mode with an IC_50_ of 1.6 µM. Furthermore, NS8593 suppresses TRPM7-dependent motility of HEK293 cells [80]. Epithelial–mesenchymal transition (EMT) in breast cancer cells is a Ca^2+^ dependent processes. Studies based on an RNA_i_ silencing approach in combination with NS8593 highlighted a role of the TRPM7 channel in this process [83]. Recently, Siddiqui *et al.* [84] took advantage of NS8593 and showed that TRPM7 critically contributes to the ability of microglia cells to migrate and invade in anti-inflammatory states. In addition, Schilling *et al.* [85] employed NS8593 to demonstrate that the TRPM7 channel is required for proliferation and polarization of macrophages towards an anti-inflammatory phenotype.

Waixenicin A (Figure 2a), a natural terpenoid of the soft coral *Sarcothelia edmondsoni* inactivated TRPM7 currents in an Mg^2+^ dependent manner with an IC_50_ of 7 µM in the absence of internal Mg^2+^ [79]. Moreover, waixenicin A was found to be efficient in suppression of TRPM7-dependent proliferation of RBL cells [79]. More recently, Kim *et al.* [86] employed waixenicin A to elucidate the functional role of TRPM7 in interstitial cells of Cajal and found that this terpenoid inhibits endogenous TRPM7 currents leading to a block of pacemaker activity of interstitial cells [86]. Waixenicin A also inhibits the growth and survival of the human gastric and breast adenocarcinoma cells (AGS and MCF-7, respectively) suggesting that TRPM7 may turn out to be a novel therapeutic target in gastric and breast cancer [86]. Yet, other researchers [87] used waixenicin A to demonstrate that TRPM7 regulates actomyosin contractility and invadosome formation in N1E-115 mouse neuroblastoma cells.

Sphingosine, the core building block of sphingolipids in the plasma membrane and its synthetic homolog FTY720 (Figure 2a) inactivated the TRPM7 channel with IC_50_’s of 0.6 µM and 0.7 µM, respectively [81]. Sphingosine and FTY720 were able to suppress TRPM7-dependent motility of HEK293 cells [81], pacemaker activity of interstitial cells of Cajal [88], and polarization of macrophages [85]. 

To summarize, several potent inhibitors of the TRPM7 channel with IC_50_ values in the low µM range have been identified. Pharmacological targeting in conjunction with genetic silencing of TRPM7 or comparative analysis of effects induced by structurally unrelated TRPM7 blockers are promising experimental strategies to uncover hitherto unrecognized cellular functions of TRPM7. 

## 3. Drug-Like Compounds Acting as Activators of the TRPM7 Channel

Recently our group has identified a set of small molecules serving as TRPM7 channel agonists [21]. We implemented a Ca^2+^ imaging-based assay to screen for activators of recombinant TRPM7 and identified 20 drug-like compounds (Table 2) with different structural backbones that can stimulate TRPM7-mediated Ca^2+^ influx and TRPM7 currents [21]. Among the latter compounds, we studied naltriben (Figure 2b) in greater detail [21]. Naltriben reversibly activates recombinant and native TRPM7 channels without prior depletion of intracellular Mg^2+^ and even under conditions of low PIP_2._ The calculated EC_50_ value was about 20 µM. The stimulatory effect of 50 µM naltriben was not observed when testing several TRP channels like TRPM2, TRPM8 and TRPV1. Furthermore, we showed that naltriben interfered with the inhibitory effect of NS8593 on TRPM7 currents in a competitive fashion. Our experiments with TRPM7 variants carrying mutations in the pore, TRP and kinase domains suggested that the site of TRPM7 activation by naltriben is most likely located in the TRP domain [21]. Naltriben functions as an antagonist of δ-opioid receptors [89]. It shows high structural similarity to other broadly used opioid receptor antagonists, with naltrindole (Figure 2b) most closely resembling naltriben. Of note, we observed that neither naltrindole, nor more distantly related analogs of naltriben like naltrexone and morphine were able to induce TRPM7 currents [21]. Taken together, we proposed that naltriben represents a positive gating modulator of the TRPM7 channel.

**Table 2:** Organic compounds activating TRPM7 channel [21].

Compound	EC_50_ (μM)	Description of the Effect
Naltriben	20.7	Reversible, [Mg^2+^]_i_ independent
Clozapine	n.d	Tested only at 30–50 μM
Proadifen	n.d	Tested only at 30–50 μM
Doxepin	n.d	Tested only at 30–50 μM
A3 hydrochloride	n.d	Tested only at 30–50 μM
Mibefradil	n.d	Tested only at 30–50 μM
U-73343	n.d	Tested only at 30–50 μM
CGP-74514A	n.d	Tested only at 30–50 μM
Metergoline	n.d	Tested only at 30–50 μM
L-733,060	n.d	Tested only at 30–50 μM
A-77636	n.d	Tested only at 30–50 μM
ST-148	n.d	Tested only at 30–50 μM
Clemastine	n.d	Tested only at 30–50 μM
Desipramine	n.d	Tested only at 30–50 μM
Sertraline	n.d	Tested only at 30–50 μM
Methiothepin	n.d	Tested only at 30–50 μM
NNC 55–0396	n.d	Tested only at 30–50 μM
Prochlorperazine	n.d	Tested only at 30–50 μM
Nortriptyline	n.d	Tested only at 30–50 μM
Loperamide	n.d	Tested only at 30–50 μM

These investigations underscore significant experimental advantages of TRPM7 agonists. TRPM7 carries very small divalent cation-selective inward currents at physiological membrane potentials. Therefore, a commonly used approach to quantify TRPM7 channel activity relies on fairly large monovalent outward cation currents (usually Cs^+^) measured at artificially high positive membrane potentials (+100 mV) upon depletion of intracellular Mg^2+^. These experimental results, however, can hardly be correlated with TRPM7-mediated influx of divalent cations at physiological membrane potentials in the presence of internal Mg^2+^ and Mg-ATP. In contrast, naltriben allows for the recording of TRPM7 currents without chelation of intracellular Mg^2+^. Furthermore, naltriben is well suited to monitor TRPM7 activity using Ca^2+^ imaging techniques that are easily adaptable to screen for new TRPM7 modulators, and in experiments with freshly isolated/primary cells that are difficult to culture or problematic to assess by the patch-clamp technique. Finally, it will be interesting to study whether activation of TRPM7 currents would impact the function of the TRPM7 kinase.

## 4. Conclusions/Outlook

In the recent past, several research groups identified a set of small organic modulators of the TRPM7 channel. These research efforts resulted in new compounds allowing for the first time to probe TRPM7 currents in native tissues under physiological conditions. The identified molecules have the potential to serve as lead structures for the development of high-affinity *in vivo* drugs targeting TRPM7. Drugs specifically acting on the TRPM7 kinase are not available yet. In the future, an additional rewarding line of research will be the identification of specific drugs acting on the TRPM7 kinase moiety to decipher TRPM7 channel *versus* kinase function in cellular physiology and pathophysiology. 

## References

[1] Nadler M.J., Hermosura M.C., Inabe K., Perraud A.L., Zhu Q., Stokes A.J., Kurosaki T., Kinet J.P., Penner R., Scharenberg A.M. (2001). LTRPC7 is a Mg.ATP-regulated divalent cation channel required for cell viability. Nature.

[2] Runnels L.W., Yue L., Clapham D.E. (2001). TRP-PLIK, a bifunctional protein with kinase and ion channel activities. Science.

[3] Ryazanov A.G., Pavur K.S., Dorovkov M.V. (1999). Alpha-kinases: A new class of protein kinases with a novel catalytic domain. Curr. Biol..

[4] Yamaguchi H., Matsushita M., Nairn A.C., Kuriyan J. (2001). Crystal structure of the atypical protein kinase domain of a trp channel with phosphotransferase activity. Mol. Cell.

[5] Fleig A., Chubanov V. (2014). TRPM7. Handb. Exp. Pharmacol..

[6] Schlingmann K.P., Waldegger S., Konrad M., Chubanov V., Gudermann T. (2007). TRPM6 and TRPM7—gatekeepers of human magnesium metabolism. Biochim. Biophys. Acta.

[7] Schlingmann K.P., Weber S., Peters M., Nejsum L.N., Vitzthum H., Klingel K., Kratz M., Haddad E., Ristoff E., Dinour D. (2002). Hypomagnesemia with secondary hypocalcemia is caused by mutations in TRPM6, a new member of the TRPM gene family. Nat. Genet..

[8] Walder R.Y., Landau D., Meyer P., Shalev H., Tsolia M., Borochowitz Z., Boettger M.B., Beck G.E., Englehardt R.K., Carmi R. (2002). Mutation of TRPM6 causes familial hypomagnesemia with secondary hypocalcemia. Nat. Genet..

[9] Chubanov V., Waldegger S., y Schnitzler M.M., Vitzthum H., Sassen M.C., Seyberth H.W., Konrad M., Gudermann T. (2004). Disruption of TRPM6/TRPM7 complex formation by a mutation in the TRPM6 gene causes hypomagnesemia with secondary hypocalcemia. Proc. Natl. Acad. Sci. USA.

[10] Ryazanov A.G. (2002). Elongation factor-2 kinase and its newly discovered relatives. FEBS Lett..

[11] Chubanov V., Gudermann T. (2014). TRPM6. Handb. Exp. Pharmacol..

[12] Penner R., Fleig A. (2007). The Mg^2+^ and Mg^2+^-nucleotide-regulated channel-kinase TRPM7. Handb. Exp. Pharmacol..

[13] Paravicini T.M., Chubanov V., Gudermann T. (2012). TRPM7: A unique channel involved in magnesium homeostasis. Int. J. Biochem. Cell Biol..

[14] Runnels L.W. (2010). TRPM6 and TRPM7: A Mul-TRP-PLIK-cation of channel functions. Curr. Pharm. Biotechnol..

[15] Bates-Withers C., Sah R., Clapham D.E. (2011). TRPM7, the Mg^2+^ inhibited channel and kinase. Adv. Exp. Med. Biol..

[16] Mederos y Schnitzler M., Waring J., Gudermann T., Chubanov V. (2008). Evolutionary determinants of divergent calcium selectivity of TRPM channels. FASEB J..

[17] Chubanov V., Schlingmann K.P., Waring J., Heinzinger J., Kaske S., Waldegger S., Mederos y Schnitzler M., Gudermann T. (2007). Hypomagnesemia with secondary hypocalcemia due to a missense mutation in the putative pore-forming region of TRPM6. J. Biol. Chem..

[18] Li M., Du J., Jiang J., Ratzan W., Su L.T., Runnels L.W., Yue L. (2007). Molecular determinants of Mg^2+^ and Ca^2+^ permeability and pH sensitivity in TRPM6 and TRPM7. J. Biol. Chem..

[19] Hofmann T., Chubanov V., Gudermann T., Montell C. (2003). TRPM5 is a voltage-modulated and Ca^2+^-activated monovalent selective cation channel. Curr. Biol..

[20] Kaske S., Krasteva G., Konig P., Kummer W., Hofmann T., Gudermann T., Chubanov V. (2007). TRPM5, a taste-signaling transient receptor potential ion-channel, is a ubiquitous signaling component in chemosensory cells. BMC Neurosci..

[21] Hofmann T., Schafer S., Linseisen M., Sytik L., Gudermann T., Chubanov V. (2014). Activation of TRPM7 channels by small molecules under physiological conditions. Pflugers. Arch..

[22] Xie J., Sun B., Du J., Yang W., Chen H.C., Overton J.D., Runnels L.W., Yue L. (2011). Phosphatidylinositol 4,5-bisphosphate (pip(2)) controls magnesium gatekeeper TRPM6 activity. Sci. Rep..

[23] Kaitsuka T., Katagiri C., Beesetty P., Nakamura K., Hourani S., Tomizawa K., Kozak J.A., Matsushita M. (2014). Inactivation of TRPM7 kinase activity does not impair its channel function in mice. Sci. Rep..

[24] Schmitz C., Perraud A.L., Johnson C.O., Inabe K., Smith M.K., Penner R., Kurosaki T., Fleig A., Scharenberg A.M. (2003). Regulation of vertebrate cellular Mg^2+^ homeostasis by TRPM7. Cell.

[25] Ryazanova L.V., Rondon L.J., Zierler S., Hu Z., Galli J., Yamaguchi T.P., Mazur A., Fleig A., Ryazanov A.G. (2010). TRPM7 is essential for Mg^2+^ homeostasis in mammals. Nat. Commun..

[26] Sahni J., Scharenberg A.M. (2008). TRPM7 ion channels are required for sustained phosphoinositide 3-kinase signaling in lymphocytes. Cell Metab..

[27] Su L.T., Agapito M.A., Li M., Simonson W.T., Huttenlocher A., Habas R., Yue L., Runnels L.W. (2006). TRPM7 regulates cell adhesion by controlling the calcium-dependent protease calpain. J. Biol. Chem..

[28] Wei C., Wang X., Chen M., Ouyang K., Song L.S., Cheng H. (2009). Calcium flickers steer cell migration. Nature.

[29] Clark K., Langeslag M., van Leeuwen B., Ran L., Ryazanov A.G., Figdor C.G., Moolenaar W.H., Jalink K., van Leeuwen F.N. (2006). TRPM7, a novel regulator of actomyosin contractility and cell adhesion. EMBO J..

[30] Meng X., Cai C., Wu J., Cai S., Ye C., Chen H., Yang Z., Zeng H., Shen Q., Zou F. (2013). TRPM7 mediates breast cancer cell migration and invasion through the MAPK pathway. Cancer Lett..

[31] Siddiqui T.A., Lively S., Vincent C., Schlichter L.C. (2012). Regulation of podosome formation, microglial migration and invasion by Ca^2+^-signaling molecules expressed in podosomes. J. Neuroinflam..

[32] Kuras Z., Yun Y.H., Chimote A.A., Neumeier L., Conforti L. (2012). KCA3.1 and TRPM7 channels at the uropod regulate migration of activated human T cells. PLoS One.

[33] Su L.T., Liu W., Chen H.C., Gonzalez-Pagan O., Habas R., Runnels L.W. (2011). TRPM7 regulates polarized cell movements. Biochem. J..

[34] Chen J.P., Luan Y., You C.X., Chen X.H., Luo R.C., Li R. (2010). TRPM7 regulates the migration of human nasopharyngeal carcinoma cell by mediating Ca^2+^ influx. Cell Calcium.

[35] Chen K.H., Xu X.H., Liu Y., Hu Y., Jin M.W., Li G.R. (2013). TRPM7 channels regulate proliferation and adipogenesis in 3T3-L1 preadipocytes. J. Cell. Physiol..

[36] Zhang Z., Wang M., Fan X.H., Chen J.H., Guan Y.Y., Tang Y.B. (2012). Upregulation of TRPM7 channels by angiotensin II triggers phenotypic switching of vascular smooth muscle cells of ascending aorta. Circ. Res..

[37] Abed E., Martineau C., Moreau R. (2011). Role of melastatin transient receptor potential 7 channels in the osteoblastic differentiation of murine MC3T3 cells. Calcif Tissue Int..

[38] Numata T., Shimizu T., Okada Y. (2007). Direct mechano-stress sensitivity of TRPM7 channel. Cell. Physiol. Biochem..

[39] Oancea E., Wolfe J.T., Clapham D.E. (2006). Functional TRPM7 channels accumulate at the plasma membrane in response to fluid flow. Circ. Res..

[40] Brauchi S., Krapivinsky G., Krapivinsky L., Clapham D.E. (2008). TRPM7 facilitates cholinergic vesicle fusion with the plasma membrane. Proc. Natl. Acad. Sci. USA.

[41] Aarts M., Iihara K., Wei W.L., Xiong Z.G., Arundine M., Cerwinski W., MacDonald J.F., Tymianski M. (2003). A key role for TRPM7 channels in anoxic neuronal death. Cell.

[42] Touyz R.M. (2008). Transient receptor potential melastatin 6 and 7 channels, magnesium transport, and vascular biology: Implications in hypertension. Am J. Physiol. Heart Circ. Physiol..

[43] Hermosura M.C., Nayakanti H., Dorovkov M.V., Calderon F.R., Ryazanov A.G., Haymer D.S., Garruto R.M. (2005). A TRPM7 variant shows altered sensitivity to magnesium that may contribute to the pathogenesis of two guamanian neurodegenerative disorders. Proc. Natl. Acad. Sci. USA.

[44] Tseveleki V., Rubio R., Vamvakas S.S., White J., Taoufik E., Petit E., Quackenbush J., Probert L. (2010). Comparative gene expression analysis in mouse models for multiple sclerosis, alzheimer’s disease and stroke for identifying commonly regulated and disease-specific gene changes. Genomics.

[45] Du J., Xie J., Zhang Z., Tsujikawa H., Fusco D., Silverman D., Liang B., Yue L. (2010). TRPM7-mediated Ca^2+^ signals confer fibrogenesis in human atrial fibrillation. Circ. Res..

[46] Guilbert A., Gautier M., Dhennin-Duthille I., Haren N., Sevestre H., Ouadid-Ahidouch H. (2009). Evidence that TRPM7 is required for breast cancer cell proliferation. Am. J. Physiol. Cell Physiol..

[47] Kim B.J., Park E.J., Lee J.H., Jeon J.H., Kim S.J., So I. (2008). Suppression of transient receptor potential melastatin 7 channel induces cell death in gastric cancer. Cancer Sci..

[48] Jiang J., Li M.H., Inoue K., Chu X.P., Seeds J., Xiong Z.G. (2007). Transient receptor potential melastatin 7-like current in human head and neck carcinoma cells: Role in cell proliferation. Cancer Res..

[49] Hanano T., Hara Y., Shi J., Morita H., Umebayashi C., Mori E., Sumimoto H., Ito Y., Mori Y., Inoue R. (2004). Involvement of TRPM7 in cell growth as a spontaneously activated Ca^2+^ entry pathway in human retinoblastoma cells. J. Pharmacol. Sci..

[50] Middelbeek J., Kuipers A.J., Henneman L., Visser D., Eidhof I., van Horssen R., Wieringa B., Canisius S.V., Zwart W., Wessels L.F. (2012). TRPM7 is required for breast tumor cell metastasis. Cancer Res..

[51] Rybarczyk P., Gautier M., Hague F., Dhennin-Duthille I., Chatelain D., Kerr-Conte J., Pattou F., Regimbeau J.M., Sevestre H., Ouadid-Ahidouch H. (2012). Transient receptor potential melastatin-related 7 channel is overexpressed in human pancreatic ductal adenocarcinomas and regulates human pancreatic cancer cell migration. Int. J. Cancer.

[52] Chen Y.F., Chen Y.T., Chiu W.T., Shen M.R. (2013). Remodeling of calcium signaling in tumor progression. J. Biomed. Sci..

[53] Gao H., Chen X., Du X., Guan B., Liu Y., Zhang H. (2011). EGF enhances the migration of cancer cells by up-regulation of TRPM7. Cell Calcium.

[54] Arking D.E., Pulit S.L., Crotti L., van der Harst P., Munroe P.B., Koopmann T.T., Sotoodehnia N., Rossin E.J., Morley M., Wang X. (2014). Genetic association study of QT interval highlights role for calcium signaling pathways in myocardial repolarization. Nat. Genet..

[55] Jin J., Desai B.N., Navarro B., Donovan A., Andrews N.C., Clapham D.E. (2008). Deletion of TRPM7 disrupts embryonic development and thymopoiesis without altering Mg^2+^ homeostasis. Science.

[56] Elizondo M.R., Arduini B.L., Paulsen J., MacDonald E.L., Sabel J.L., Henion P.D., Cornell R.A., Parichy D.M. (2005). Defective skeletogenesis with kidney stone formation in dwarf zebrafish mutant for TRPM7. Curr. Biol..

[57] Jin J., Wu L.J., Jun J., Cheng X., Xu H., Andrews N.C., Clapham D.E. (2012). The channel kinase, TRPM7, is required for early embryonic development. Proc. Natl. Acad. Sci. USA.

[58] Sah R., Mesirca P., Van den Boogert M., Rosen J., Mably J., Mangoni M.E., Clapham D.E. (2013). Ion channel-kinase TRPM7 is required for maintaining cardiac automaticity. Proc. Natl. Acad. Sci. USA.

[59] Sah R., Mesirca P., Mason X., Gibson W., Bates-Withers C., Van den Boogert M., Chaudhuri D., Pu W.T., Mangoni M.E., Clapham D.E. (2013). Timing of myocardial TRPM7 deletion during cardiogenesis variably disrupts adult ventricular function, conduction, and repolarization. Circulation.

[60] Dorovkov M.V., Ryazanov A.G. (2004). Phosphorylation of annexin I by TRPM7 channel-kinase. J. Biol. Chem..

[61] Clark K., Middelbeek J., Lasonder E., Dulyaninova N.G., Morrice N.A., Ryazanov A.G., Bresnick A.R., Figdor C.G., van Leeuwen F.N. (2008). TRPM7 regulates myosin IIA filament stability and protein localization by heavy chain phosphorylation. J. Mol. Biol..

[62] Perraud A.L., Zhao X., Ryazanov A.G., Schmitz C. (2011). The channel-kinase TRPM7 regulates phosphorylation of the translational factor EEF2 via EEF2-K. Cell Signal.

[63] Deason-Towne F., Perraud A.L., Schmitz C. (2012). Identification of ser/thr phosphorylation sites in the C2-domain of phospholipase c gamma2 (plcgamma2) using TRPM7-kinase. Cell Signal.

[64] Clark K., Middelbeek J., Morrice N.A., Figdor C.G., Lasonder E., van Leeuwen F.N. (2008). Massive autophosphorylation of the ser/thr-rich domain controls protein kinase activity of TRPM6 and TRPM7. PLoS One.

[65] Matsushita M., Kozak J.A., Shimizu Y., McLachlin D.T., Yamaguchi H., Wei F.Y., Tomizawa K., Matsui H., Chait B.T., Cahalan M.D. (2005). Channel function is dissociated from the intrinsic kinase activity and autophosphorylation of TRPM7/CHAK1. J. Biol. Chem..

[66] Desai B.N., Krapivinsky G., Navarro B., Krapivinsky L., Carter B.C., Febvay S., Delling M., Penumaka A., Ramsey I.S., Manasian Y. (2012). Cleavage of TRPM7 releases the kinase domain from the ion channel and regulates its participation in fas-induced apoptosis. Dev. Cell.

[67] Krapivinsky G., Krapivinsky L., Manasian Y., Clapham D.E. (2014). The TRPM7 chanzyme is cleaved to release a chromatin-modifying kinase. Cell.

[68] Monteilh-Zoller M.K., Hermosura M.C., Nadler M.J., Scharenberg A.M., Penner R., Fleig A. (2003). TRPM7 provides an ion channel mechanism for cellular entry of trace metal ions. J. Gen. Physiol..

[69] Demeuse P., Penner R., Fleig A. (2006). TRPM7 channel is regulated by magnesium nucleotides via its kinase domain. J. Gen. Physiol..

[70] Schmitz C., Deason F., Perraud A.L. (2007). Molecular components of vertebrate mg^2+^-homeostasis regulation. Magnes. Res..

[71] Runnels L.W., Yue L., Clapham D.E. (2002). The TRPM7 channel is inactivated by pip(2) hydrolysis. Nat. Cell Biol..

[72] Kozak J.A., Matsushita M., Nairn A.C., Cahalan M.D. (2005). Charge screening by internal pH and polyvalent cations as a mechanism for activation, inhibition, and rundown of TRPM7/MIC channels. J. Gen. Physiol..

[73] Prakriya M., Lewis R.S. (2002). Separation and characterization of currents through store-operated crac channels and Mg^2+^-Inhibited Cation (MIC) channels. J. Gen. Physiol..

[74] Li M., Jiang J., Yue L. (2006). Functional characterization of homo- and heteromeric channel kinases TRPM6 and TRPM7. J. Gen. Physiol..

[75] Kozak J.A., Kerschbaum H.H., Cahalan M.D. (2002). Distinct properties of crac and mic channels in RBL cells. J. Gen. Physiol..

[76] Chen X., Numata T., Li M., Mori Y., Orser B.A., Jackson M.F., Xiong Z.G., MacDonald J.F. (2010). The modulation of TRPM7 currents by nafamostat mesilate depends directly upon extracellular concentrations of divalent cations. Mol. Brain.

[77] Parnas M., Peters M., Dadon D., Lev S., Vertkin I., Slutsky I., Minke B. (2009). Carvacrol is a novel inhibitor of drosophila trpl and mammalian TRPM7 channels. Cell Calcium.

[78] Chen H.C., Xie J., Zhang Z., Su L.T., Yue L., Runnels L.W. (2010). Blockade of TRPM7 channel activity and cell death by inhibitors of 5-lipoxygenase. PLoS One.

[79] Zierler S., Yao G., Zhang Z., Kuo W.C., Porzgen P., Penner R., Horgen F.D., Fleig A. (2011). Waixenicin a inhibits cell proliferation through magnesium-dependent block of transient receptor potential melastatin 7 (TRPM7) channels. J. Biol. Chem..

[80] Chubanov V., y Schnitzler M.M., Meissner M., Schafer S., Abstiens K., Hofmann T., Gudermann T. (2012). Natural and synthetic modulators of SK (k(ca)2) potassium channels inhibit magnesium-dependent activity of the kinase-coupled cation channel TRPM7. Br. J. Pharmacol..

[81] Qin X., Yue Z., Sun B., Yang W., Xie J., Ni E., Feng Y., Mahmood R., Zhang Y., Yue L. (2013). Sphingosine and FTY720 are potent inhibitors of the transient receptor potential melastatin 7 (TRPM7) channels. Br. J. Pharmacol..

[82] Chokshi R., Fruasaha P., Kozak J.A. (2012). 2-aminoethyl diphenyl borinate (2-apb) inhibits TRPM7 channels through an intracellular acidification mechanism. Channels (Austin).

[83] Davis F.M., Azimi I., Faville R.A., Peters A.A., Jalink K., Putney J.W., Goodhill G.J., Thompson E.W., Roberts-Thomson S.J., Monteith G.R. (2014). Induction of epithelial-mesenchymal transition (EMT) in breast cancer cells is calcium signal dependent. Oncogene.

[84] Siddiqui T., Lively S., Ferreira R., Wong R., Schlichter L.C. (2014). Expression and contributions of TRPM7 and KCA2.3/SK3 channels to the increased migration and invasion of microglia in anti-inflammatory activation states. PLoS One.

[85] Schilling T., Miralles F., Eder C. (2014). TRPM7 channels regulate proliferation and polarisation of macrophages. J. Cell Sci..

[86] Kim B.J., Nam J.H., Kwon Y.K., So I., Kim S.J. (2013). The role of waixenicin a as transient receptor potential melastatin 7 blocker. Basic Clin. Pharmacol. Toxicol..

[87] Visser D., Langeslag M., Kedziora K.M., Klarenbeek J., Kamermans A., Horgen F.D., Fleig A., van Leeuwen F.N., Jalink K. (2013). TRPM7 triggers Ca^2+^ sparks and invadosome formation in neuroblastoma cells. Cell Calcium.

[88] Nam J.H., Kim W.K., Kim B.J. (2013). Sphingosine and FTY720 modulate pacemaking activity in interstitial cells of cajal from mouse small intestine. Mol. Cells.

[89] Sofuoglu M., Portoghese P.S., Takemori A.E. (1991). Differential antagonism of delta opioid agonists by naltrindole and its benzofuran analog (NTB) in mice: Evidence for delta opioid receptor subtypes. J. Pharmacol. Exp. Ther..

